# Supplementary material for: Rapid quantification of miRNAs using dynamic FRET-FISH
Source: Commun Biol. 2022 Oct 7;5:1072. doi: 10.1038/s42003-022-04036-x (PMC9546913; doi:10.1038/s42003-022-04036-x)
Supplement: Supplementary file 2 — Supplementary Information [file 42003_2022_4036_MOESM2_ESM.pdf]

Supplementary Information

Rapid quantification of miRNAs using dynamic FRET-FISH

Juyoung Kim[1], Chanshin Kang[1], Soochul Shin[1*] & Sungchul Hohng[1*]

[1]Department of Physics and Astronomy, Institute of Applied Physics, Seoul National University, Seoul, Republic of Korea

Supplementary Fig. 1. | More representative fluorescence intensity time traces and kinetic parameters obtained using hidden Markov modeling. More than 3,000 molecules were used for data analysis.

a

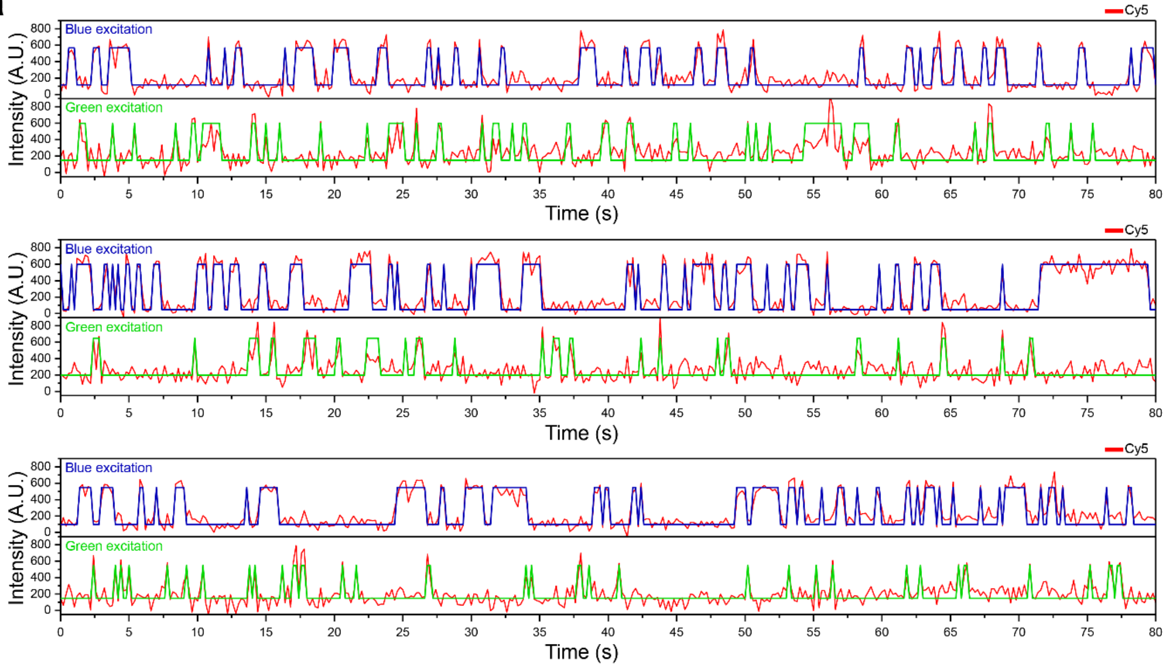

b

donor1		donor2	
k_{off}	k_{on}	k_{off}	k_{on}
1.51 (±0.06) s^{-1}	6.00 (±0.33)x10^5 $M^{-1}s^{-1}$	3.31 (±1.16) s^{-1}	4.01 (±0.09) x10^5 $M^{-1}s^{-1}$

Supplementary Fig. 2. | Design of DNA probe sets for detection of let-7 miRNA family.

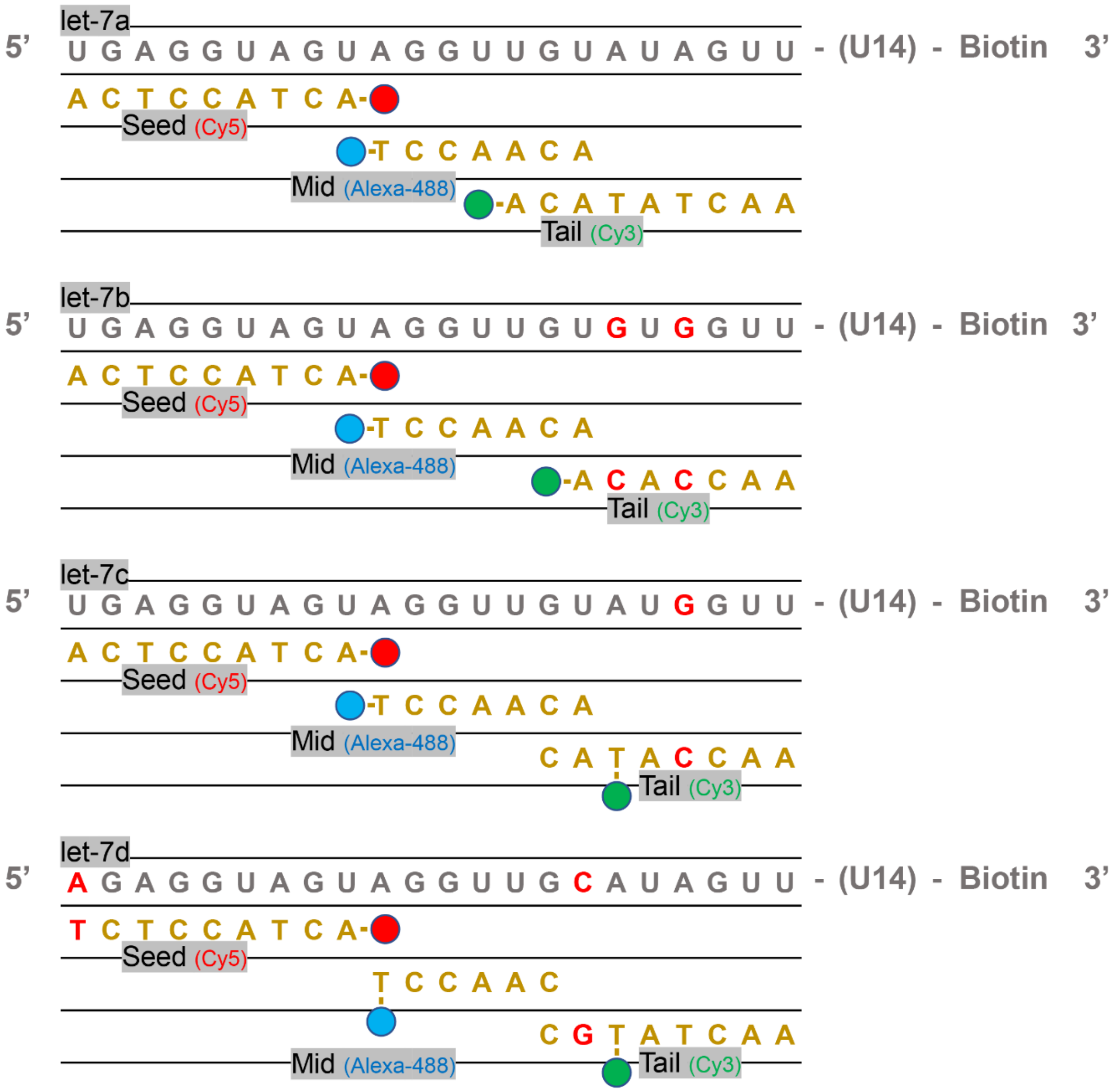

Supplementary Fig. 3. | Comparisons of target specificities of various miRNA detection techniques.

TaqMan

Relative detection (%)				
miRNA / Probe	let-7a	let-7b	let-7c	let7-d
Probe A	100	0.4	20.9	2.2
Probe B	0.2	100	22.5	0.0
Probe C	0.1	1.8	100	0.0
Probe D	2.6	0.0	1.4	100

Quanta

Relative detection (%)				
miRNA / Probe	let-7a	let-7b	let-7c	let7-d
Probe A	100	0.3	50.7	2.2
Probe B	0.1	100	32.8	0.0
Probe C	48.9	27.0	100	0.3
Probe D	0.1	0.3	0.1	100

miQPCR

Relative detection (%)				
miRNA / Probe	let-7a	let-7b	let-7c	let7-d
Probe A	100	12.6	55.5	101.8
Probe B	7.8	100	45.5	1.1
Probe C	66.4	75.1	100	28.8
Probe D	14.8	0.0	0.1	100

Ago-FISH

Relative detection (%)				
miRNA / Probe	let-7a	let-7b	let-7c	let7-d
Probe A	100	1.0	3.1	0.0
Probe B	0.0	100	3.4	0.0
Probe C	1.3	0.0	100	0.0
Probe D	0.0	0.0	5.1	100

dynamic FRET-FISH

Relative detection (%)				
miRNA / Probe	let-7a	let-7b	let-7c	let7-d
Probe A	100	1.2	1.2	0.2
Probe B	1.1	100	0.5	0.2
Probe C	0.9	1.2	100	0.1
Probe D	0.2	0.1	0.1	100
