# Supplementary material for: Rapid quantification of miRNAs using dynamic FRET-FISH
Source: Commun Biol. 2022 Oct 7;5:1072. doi: 10.1038/s42003-022-04036-x (PMC9546913; doi:10.1038/s42003-022-04036-x)
Supplement: Supplementary file 3 — Description of Additional Supplementary Files [file 42003_2022_4036_MOESM3_ESM.pdf]

Description of Additional Supplementary Files

File name: Supplementary Data 1

Description: The source data behind Figure 1d-e, 2c, 3, and S1a.
